# Next generation sequencing for molecular diagnosis of neuromuscular diseases

**DOI:** 10.1007/s00401-012-0982-8

**Published:** 2012-04-18

**Authors:** Nasim Vasli, Johann Böhm, Stéphanie Le Gras, Jean Muller, Cécile Pizot, Bernard Jost, Andoni Echaniz-Laguna, Vincent Laugel, Christine Tranchant, Rafaelle Bernard, Frédéric Plewniak, Serge Vicaire, Nicolas Levy, Jamel Chelly, Jean-Louis Mandel, Valérie Biancalana, Jocelyn Laporte

**Affiliations:** 1 IGBMC (Institut de Génétique et de Biologie Moléculaire et Cellulaire), 1, rue Laurent Fries, BP10142, 67404 Illkirch, France; 2 U964, Inserm, Illkirch, France; 3 UMR7104, CNRS, Illkirch, France; 4 Université de Strasbourg, Illkirch, France; 5 Chaire de génétique humaine, Collège de France, Illkirch, France; 6 Laboratoire Diagnostic Génétique, Faculté de Médecine, CHRU, Strasbourg, France; 7 Département de Neurologie, Hôpital Civil de Strasbourg, Strasbourg, France; 8 Service de Pédiatrie, Centre Hospitalier Universitaire (CHU), Strasbourg, France; 9 Faculté de Médecine de Marseille, Inserm UMRS 910 Génétique Médicale et Génomique Fonctionnelle, Université de la Méditerranée, Marseille, France; 10 Institut Cochin, INSERM Unité 1016, CNR UMR 1408, Université Paris Descartes, Sorbonne Paris Cité, Paris, France

**Keywords:** Neuromuscular disorder, Sequencing, Molecular diagnosis, DNA barcoding, Myopathy, Neuropathy

## Abstract

Inherited neuromuscular disorders (NMD) are chronic genetic diseases posing a significant burden on patients and the health care system. Despite tremendous research and clinical efforts, the molecular causes remain unknown for nearly half of the patients, due to genetic heterogeneity and conventional molecular diagnosis based on a gene-by-gene approach. We aimed to test next generation sequencing (NGS) as an efficient and cost-effective strategy to accelerate patient diagnosis. We designed a capture library to target the coding and splice site sequences of all known NMD genes and used NGS and DNA multiplexing to retrieve the pathogenic mutations in patients with heterogeneous NMD with or without known mutations. We retrieved all known mutations, including point mutations and small indels, intronic and exonic mutations, and a large deletion in a patient with Duchenne muscular dystrophy, validating the sensitivity and reproducibility of this strategy on a heterogeneous subset of NMD with different genetic inheritance. Most pathogenic mutations were ranked on top in our blind bioinformatic pipeline. Following the same strategy, we characterized probable *TTN*, *RYR1* and *COL6A3* mutations in several patients without previous molecular diagnosis. The cost was less than conventional testing for a single large gene. With appropriate adaptations, this strategy could be implemented into a routine genetic diagnosis set-up as a first screening approach to detect most kind of mutations, potentially before the need of more invasive and specific clinical investigations. An earlier genetic diagnosis should provide improved disease management and higher quality genetic counseling, and ease access to therapy or inclusion into therapeutic trials.

**Electronic supplementary material:**

The online version of this article (doi:10.1007/s00401-012-0982-8) contains supplementary material, which is available to authorized users.

## Introduction

Inherited neuromuscular disorders (NMD) form a group of genetic diseases which result in chronic long term disability posing a significant burden to the patients, their families and public health care. NMD are often severe and include more than 200 monogenic disorders with a total incidence exceeding 1 in 3,000 [3]. Despite tremendous research and clinical efforts, the molecular causes of NMD are still unknown for approximately half of patients. The precise diagnosis of NMD requires an extensive clinical evaluation in conjunction with targeted complementary tests. To date, routine genetic diagnosis is mainly done on a gene-by-gene basis, starting from the most pertinent one. Diagnostic challenges in this group of diseases include genetic heterogeneity in most of the disorders and lack of segregation data in sporadic cases to orient the screening. As an example, hereditary sensorimotor neuropathies (HSMN) are due to mutations in nearly 50 genes, while congenital myopathies implicate at least 14 different genes [21, 22]. Also, large genes, such as Titin (*TTN*) with 363 exons, are not entirely sequenced even if previously linked to NMD [1]. As a consequence, clinical tests are multiplied, DNA is sent to different laboratories and patients are submitted to thorough examination that includes sometimes invasive investigations. Often genetic diagnosis is delayed, exposing the patient to unnecessary investigations and treatments, precluding the full benefit of a targeted approach to treatment, and increasing recurrence risk in the families.

Current molecular diagnostic approaches are time-consuming and expensive. Recently, massively parallel sequencing using next generation sequencing (NGS) technologies has emerged as a successful approach to interrogate multiple genes simultaneously and is currently mainly used to identify novel disease genes in a research setting [10, 18–20, 31]. A fewer studies reported the use of whole genome (WGS) or whole exome (WES) sequencing for genetic diagnosis of a given monogenic disease. Concerning NMD, Lupski et al. [14] and Montenegro et al. [16], respectively, used WGS and WES in patients with hereditary sensorimotor neuropathies (HSMN) on a research setting. In both cases, they analyzed a single family and focused their variants ranking only on known HSMN genes. Targeted resequencing of known disease genes appears more relevant for routine diagnosis and until now was tested on a few specific disease genes like 5 ataxia genes or 21 breast cancer genes or a single large gene like *DMD* in very homogeneous patient cohorts, or for carrier testing [6, 13, 30].

As no previous large scale sequencing study targeting several NMD genes was reported, our aim here is to pilot an efficient screening strategy in an attempt to improve the clinical and molecular investigations of neuromuscular diseases from a very heterogeneous panel of patients. We used targeted enrichment of 267 known NMD genes followed by NGS in patients affected by different neuromuscular diseases with or without known mutations. DNA multiplexing and blind variant ranking retrieved successfully different mutation types for diseases with different segregations.

## Patients and methods

### Patients

Two groups of patients with various neuromuscular diseases were selected: eight patients with pathogenic mutations previously identified by conventional Sanger sequencing of candidate genes (patients A to H), and eight random patients without known mutations and different clinical diagnosis encompassing myopathies and neuropathies (patients I to P). Clinical and segregation data are listed in the online resource data. DNA was extracted from venous blood by three different methods: two manual methods, FlexiGene DNA kit (Qiagen GmbH, Hilden, Germany) and Bacc Nucleon 3 (Amersham-Bioscience), and one automated method, the QIA symphony DNA midi kit (Qiagen GmbH, Hilden, Germany). Informed consent was obtained from all individuals, and the study was approved by the comité de protection des personnes (DC-2012-1497).

### Targeted massively parallel sequencing

All the 267 NMD genes, known to be implicated in 16 different disease classes (online resource Table 1; http://www.musclegenetable.org/[8]) were targeted for enrichment. Capture design was done using the Agilent eArray (http://earray.chem.agilent.com/earray/). In this pilot study, we included the 267 genes because genetic heterogeneity exists in all disease classes and several genes are implicated in different classes (e.g. *LMNA* or *DNM2*). Oligonucleotides covered all coding exons and all intron–exon boundaries including at least 50 intronic nucleotides. 5′ and 3′ UTRs and deep intronic sequences were not targeted to avoid increasing the sequence target size that would have strongly decreased the mean sequence coverage. After masking the repetitive elements, the 4,604 targeted exons represented 1.6 Mb (online resource Table 2). A minimum of 3 μg of genomic DNA was sheared to obtain a mean fragment size of 250 nt using Covaris E210 (KBioscience, Herts, UK) followed by automatic library preparation with the SPRI-TE Nucleic Acid Extractor (Beckman Coulter Inc, Brea, CA) using the SPRIworks fragment library cartridge (Beckman Coulter Inc, Brea, CA) and Illumina adapters. Targeted regions were captured using the Agilent SureSelect custom target enrichment kit (Agilent Technologies, Santa Clara, CA) following Agilent protocols. Enriched DNA fragments were barcoded with the Illumina multiplexing sample preparation kit (Illumina, San Diego, CA), pooled by 4, and sequenced on an Illumina Genome Analyzer IIx to generate 72nt paired-end reads for 4 DNAs per channel, following the manufacturer’s protocols.

### Bioinformatic analysis

**Fig. 1:**
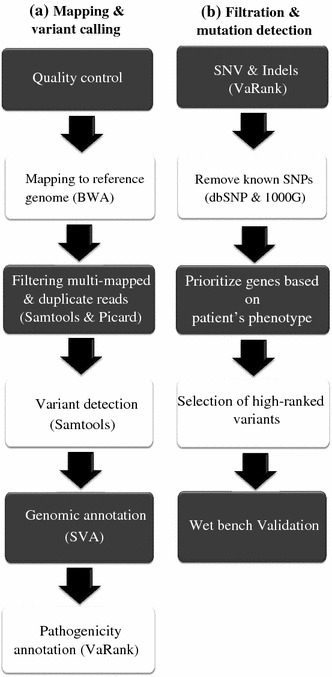
Bioinformatic filtering and ranking

The authors implicated in the sequencing and bioinformatic analysis had no information on the patient data, except for the disease class and potential segregation. The bioinformatic analysis pipeline is depicted in Fig. 1. Image analysis and base calling were performed using the Illumina Pipeline RTA (Real-Time Analysis) version 1.9. DNA sequences were aligned to the reference genome GRCh37/hg19 using BWA [11]. Insertions or deletions of up to 50nt were allowed for the alignment to the genome. Reads that mapped to several positions in the genome and reads sharing the same start position and strand were filtered out using Picard (http://picard.sourceforge.net/) and Samtools [12]. From an average of 14 million mapped reads, about 4 million were uniquely mapped in targeted regions. Valid variants had to be seen in both directions with at least 3× coverage and their calling was done using Samtools; minimum mapping quality was 25, consensus quality was 20 and minimum SNV (single nucleotide variation)/indel quality was 20. Variants were defined as homozygous, if present in more than 80 % of the reads. For SNV/indel annotation SVA (v1.02) [4] (http://www.svaproject.org/), Ensembl60 and dbSNP134 were used, and validated non-pathogenic variants present in dbSNP and 1000Genomes databases were removed.

Variants filtering and ranking were done using the VaRank program. Briefly, for each variant, VaRank used the Alamut software (Interactive Biosoftware, Rouen, France) to collect genomic annotations and different scores such as the coding status, the nucleotide and amino acid conservation scores and the effect of each change on the protein and splice site, and then compiles them to rank SNVs and indels starting from the most probable pathogenic. The SNVs/indels are characterized using several genomic or functional annotations that VaRank summarizes into a score to produce a list of ranked variants (manuscript in preparation). The probable mutations are ranked starting from the most likely to be pathogenic according to the following list: nonsense, frameshift, essential splice site (affecting the conserved consensus intronic positions), start loss, stop loss, missense, predicted splice site mutation (outside of the consensus sites), in frame indels, and synonymous coding. The scores are modulated according to the genomic conservation based on the phastcons score [7] and to the SIFT [17] and PolyPhen v2 [23] scores to assess the effect of amino acid change on the protein function. Synonymous coding variants might have an effect on the biosynthesis of the protein [28], and their potential impact on splicing was also scored. Splicing effect is assessed using three different softwares: Human Splicing Finder [2], MaxEntScan [32] and NNSplice [24]. Scores of compound heterozygous mutations in the same gene were added in case of a recessive segregation of the disease to prioritize the best candidate genes. Clinical significance was extracted from dbSNP134 and some of the known mutations were confirmed using locus-specific mutation data bases (LSDB-http://www.umd.be/). The “Clinical significance” field from dbSNP highlights known mutations with an “rs” identifier. Variants annotated as “probably-pathogenic” or “pathogenic” usually corresponded to reported mutations and were weighted to reach a high VaRank score. Indeed, these variants were not filtered as some healthy people are carriers of mutations. In the next step, genes within the patient disease class(es) were extracted and the mode of inheritance of the disease in the family, if known, was matched to the known type of transmission for every selected candidate genes.

In order to detect large deletions, a coverage-based method was used where the number of reads in a sliding window of 20nt was computed across the genome for each patient and coverage compared to three randomly selected patients.

### Mutation validation

Sanger sequencing was performed to confirm sequence variants in the original DNA samples and to assess the segregation in the families included in this study (GATC Biotech).

## Results

### Sequencing results

**Fig. 2:**
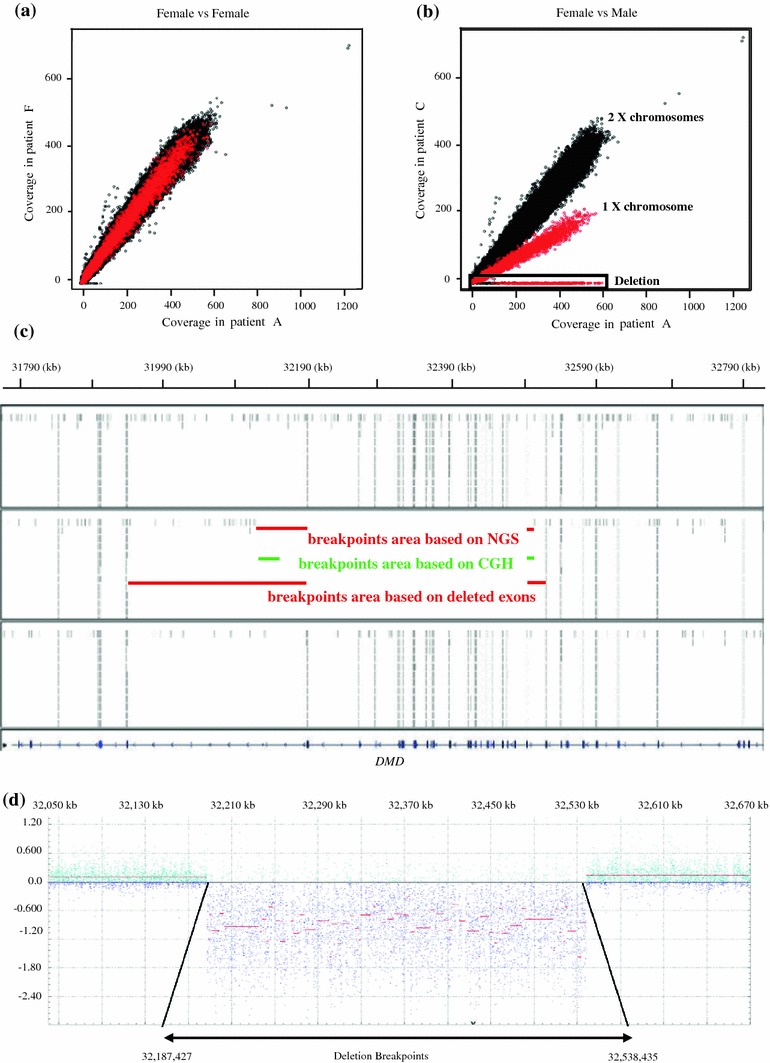
Detection of copy number and mapping of a deletion in patient C with DMD. **a**, **b** Gender determination: comparison of sequence reads mapping to the X chromosome between two female DNAs in (**a**) and a female (*black*) and a male (*red*) in (**b**). In **b** a deletion of several exons is detected on the X chromosome for the male (*squared*). **c** Next generation sequencing data showing the detection of a 27 exons deletion in patient C with DMD (*middle panel*) compared to two other DNAs (*top and bottom panels*). Random off-target reads allow a more precise mapping of the deletion breakpoints. Off-target reads varied between two different experiments. **d** CGH-array results showing the 5′ and 3′ breakpoints map between 32,538,435 and 32,538,443 and between 32,187,417 and 32,187,427, respectively

Following DNA barcoding and pooling by group of 4, targeted sequencing of the 267 known NMD genes (online resource Table 1) was performed in 8 individuals (A to H) with different neuromuscular disorders and known mutations. After alignment with the human reference genome, mean coverage of the targeted exons was 138× and the percentage of nucleotides with at least 10× coverage was 94 (Table 1, online resource Table 2). Average enrichment for targeted exons was 1,410 fold. More than 97 % of the targeted exons were fully covered, while 168 targeted exons were covered <3× in at least half of the patients (online resource Fig. 1 a and online resource Table 2). Most low-covered exons were similar between patients and coverage decreased with increasing GC content (online resource Fig. 1b). Similar findings were obtained in an independent experiment with six of these DNA samples sequenced individually, validating the multiplexing approach (data not shown). DNAs prepared with different extraction protocols gave similar results, supporting the use of these different protocols on a routine diagnosis basis. Reproducibility between different DNAs treated in the same experiment was such that the coverage was similar for given targeted exons (online resource Table 2 and online resource Fig. 2; narrow distribution of the 95th percentile close to the median). This allowed the detection of the copy number to unambiguously determine the gender of patients as a control for the experiment (Fig. 2a, b), and the mapping of a large deletion (see below).

**Table 1:** Sequencing, coverage and variant statistics

	Samples	A	B	C	D	E	F	G	H	Average
Sequencing	Sequenced nucleotides	1179572688	1143805104	749145888	1057816944	1281453552	921756240	912120912	913302000	1019871666
	Sequence after filtering (in nt)^b^	399768120	392297112	314103240	246193344	355696416	351273528	404841528	210318840	334311516
	Sequence in target regions (in nt)	316579968	321670656	249903072	193743144	271231344	274046688	220871448	169312104	252169803
Coverage	Mean coverage (x)	172	176	137	106	148	150	120	96	138
	Median coverage (x)	165	162	126	105	145	144	113	91	131
	% Base ≥3× coverage	98	97	97	98	98	98	97	97	98
	% Base ≥10× coverage	95	94	92	94	95	94	93	93	94
	Fully covered exons	4,477	4,445	4,396	4,443	4,484	4,445	4,429	4,425	4,443
Variants	SNVs	1,097	1,096	1,015	1,148	1,374	1,315	1,127	1,120	1,162
	Indels	208	148	127	139	168	153	127	146	152
	Total heterozygotes	1,018	920	779	973	1,141	1,095	910	859	962
	Total homozygotes	287	324	363	314	401	373	344	407	352
	SNVs + indels	1,305	1,244	1,142	1,287	1,542	1,468	1,254	1,266	1,314
	SNVs + indels without rs number	374	325	259	294	457	403	335	279	341
	Novel coding non-synonymous^a^	111/0	109/0	93/0	99/0	145/0	155/1	116/0	94/1	115/n.a.^c^
	Novel splice site change^a^	10/0	6/0	4/0	4/1	17/0	6/0	5/1	2/0	7/n.a.^c^
	Novel coding stop (gained/lost)^a^	3/0	4/1	0/0	0/0	0/0	1/0	1/0	1/0	1/n.a. ^c^
	Novel coding frameshift^a^	3/1	1/0	1/large deletion	2/1	3/1	1/0	2/0	1/1	2/n.a.^c^

Average enrichment is 1,410 fold

^a^Before/after filtering and ranking

^b^After filtering duplicate reads and multiple genomic mapping

^c^Not applicable

### Variants identification and ranking

Variants were identified based on the bioinformatic analysis encompassing sequence mapping, variant calling and filtering, and variant ranking (Fig. 1). On average, we found 1,162 SNVs and 152 indels of which 341 were not reported as SNP (Table 1). 125 variants affecting the essential splice sites or predicted to change the amino acid sequence were found on average in the 267 NMD genes. For prioritization, these variants were ranked using a novel scoring program (VaRank; see methods), then by extracting the different genes fitting the disease class(es) based on the general clinical phenotype of patients, and lastly based on the segregation if known (Table 2 and online resource Table 3). For patients with phenotypes that matched to several disease classes, all genes fitting the different diseases classes were considered.

**Table 2:** Mutations identified in patients with known mutations and probable mutations in patients without previous molecular characterization

Patient	Gender	Disease (segregation)	Disease class	Gene	Mutation nucleotide (protein)	Haplotype
A	F	Carrier for myotubular myopathy (XL)	Congenital myopathies	*MTM1*	Exon4: c.141–144delAAAG (p.Glu48LeufsX24)	Heterozygous
B	M	Centronuclear myopathy (AR)	Congenital myopathies	*BIN1*	Exon20: c.1717 C > T (p.Gln573X)	Homozygous
C	M	Duchenne muscular dystrophy (XL)	Muscular dystrophies	*DMD*	Deletion ex18-44	Hemizygous
D	F	Ataxia ocular apraxia (AR)	Hereditary ataxias	*SETX*	Exon10: c.3213–3214insT (p.Gln1072SerfsX3); Int10: c.5275-1 G > A	Compound heterozygous
E	M	Myotubular myopathy (XL)	Congenital myopathies	*MTM1*	Exon4: c.156–157insA (p.Cys53MetfsX8)	Hemizygous
F	F	Centronuclear myopathy (AD)	Congenital myopathies	*DNM2*	Exon14: c.1565G>A (p.Arg522His)	Heterozygous
G	M	Myotubular myopathy (XL)	Congenital myopathies	*MTM1*	Int11: c.1261–10A>G	Hemizygous
H	F	Ataxia ocular apraxia (AR)	Hereditary ataxias	*SETX*	Exon10: c.2967-2971delGAAAG(p.Arg989SerfsX5); Exon8: c.994C>T (p.Arg332Trp)	Compound heterozygous
I	M	HMSN, demyelinating CMT neuropathy (AR)^a^	n.a.^a^	None		
J	M	Myopathy with cytoplasmic aggregates	All myopathies	*TTN* ^c^	Exon292: c.68576C>T (p.Pro22859Leu)	Heterozygous
K	F	Bethlem dystrophy or myofibrillar myopathy (AD)	Muscular dystrophies, other myopathies	*COL6A3* ^c^	Exon27: c.6812G>A (p.Arg2271Lys)	Heterozygous
L	M	Hereditary spastic paraplegia (sporadic)	Hereditary paraplegias	None		
M	M	Vacuolar myopathy (sporadic)	Congenital myopathies, distal myopathies, other myopathies	None		
N	M	HMSN, axonal CMT (AR)	Hereditary neuropathies	None (LMNA)	Exon11: c.1928C>A (p.Thr643Asn); c.1930C>T (p.Arg644Cys)^b^	Compound heterozygous
O	F	Muscular dystrophy (AR)	Muscular dystrophies	*TTN* ^c^	Exon18: c.3100G>A (p.Val1034Met); Exon240: c.49243G>A (p.Ala16415Thr)	Compound heterozygous
P	M	Muscular dystrophy and arthrogryposis (AR)	Muscular dystrophies, congenital myopathies, other NMD diseases	*RYR1* ^c^	Exon55: c.8554C>T (p.Arg2852X); Exon81: c.11557G>A (p.Glu3853Lys)	Compound heterozygous

^a^ *HMSN* hereditary motor and sensory neuropathy, *CMT* Charcot–Marie–Tooth; patient I was later re-diagnosed as having a mitochondrial disease for which genes were not targeted

^b^Previously reported as pathogenic; probable monoallelic compound heterozygous

^c^Confirmed by segregation analysis

### Identification and confirmation of mutations

**Fig. 3:**
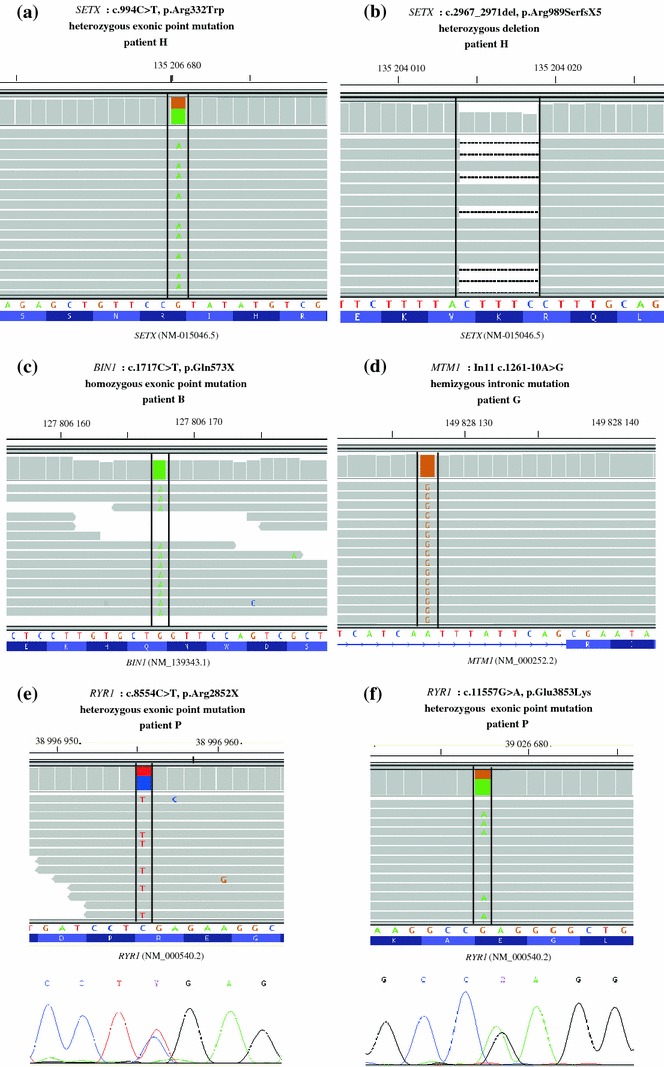
Detection of different types of mutations from patients with previously known and unknown molecular diagnosis. Compound heterozygous exonic point mutation (**a**) and heterozygous indel mutation (**b**) in the *SETX* gene in patient H with ataxia. **c** Homozygous exonic point mutation in the *BIN1* gene in patient B with centronuclear myopathy. **d** Intronic mutation in the *MTM1* gene in patient G with myotubular myopathy. **e**, **f** Novel compound heterozygous mutations detected in patient P with muscular dystrophy and arthrogryposis in the *RYR1* gene by next generation sequencing and confirmed by Sanger sequencing. Displayed with the integrative genomics viewer IGV [25]. The normal nucleotide and protein sequences are depicted at the *bottom*

We retrieved all ten different known mutations in the eight analyzed DNAs (Table 2; patients A–H). Sequencing data sustaining the mutations are depicted in (Fig. 3a–d and online resource Fig. 4). In particular, we detected homozygous and heterozygous mutations validating the detection of both alleles, point mutations or small insertion or deletions, intronic and exonic mutations. Compound heterozygous mutations in *SETX* were retrieved in the two patients with ataxia (Fig. 3 and online resource Fig. 3). Importantly, our VaRank scoring program blindly ranked the known mutations and implicated genes first in the list when taking into account the disease class and inheritance for most patients (online resource Table 3). Although the clinical data are important to define the disease class, we did not prioritize genes within each disease class based on more detailed pathological data (e.g. even if patient E had myotubular myopathy, all congenital myopathy genes were ranked), suggesting that this approach may be proposed for patients prior to extensive histological investigations. Moreover, the patients in our cohort were affected by diverse diseases of different segregation (X-linked, autosomal recessive or dominant; Table 2), validating this approach for a wide range of heterogeneous diseases and mutation types.

The large deletion encompassing exons 18–44 of the *DMD* gene was detected in a patient with Duchenne Muscular Dystrophy by comparing the number of reads in these regions with other sequenced DNA samples (Fig. 2b). The mean coverage for exons 18–44 is 0 for this patient and 177 for other patients (online resource Table 2, *DMD* gene for patient C). Unexpectedly, off-target reads from genomic DNA fragments not targeted for enrichment and located in *DMD* introns allowed to restrict the areas containing the upstream and downstream breakpoints from 27 to 11 Kb and 248 to 72 Kb, respectively (Fig. 2c). To assess the accuracy of deletion breakpoints predicted through off-target reads with the precise deleted positions, we analyzed this *DMD* deletion using the custom-designed oligonucleotides CGH-array previously described by Saillour et al. [27] where oligonucleotide probes cover both intronic and exonic *DMD* regions with an average tiling interval of 50 bases. CGH-array indicated that the 3′ breakpoint maps between 32,187,417 (position of the non-deleted probe) and 32,187,427 (position of the deleted probe), and the 5′ breakpoint maps between 32,538,435 and 32,538,443 (Fig. 2d). NGS data are coherent with CGH-array as the off-target reads closer to the deletion mapped at positions 32,072,428 and 32,547,130. The differences between the precise positions based on CGH-array and breakpoints found by NGS data are 115 kb for the 3′ breakpoint and 9 kb for the 5′ breakpoint. For a better precision of NGS method for mapping intronic breakpoints, targeting for enrichment of intronic sequences could be a possibility, but will increase the total targeted sequence length and thus decrease the overall coverage for a given sequencing depth.

Following a similar strategy, we analyzed 8 DNAs (I to P) from patients with heterogeneous neuromuscular disorders without molecular characterization. These patients were not selected based neither on the amount or quality of clinical data nor on the availability of DNA from other members of the family, to mimic the situation of routine diagnosis. There were no specific inclusion criteria. We identified probable disease-causing mutations in several patients. Sanger sequencing was used to validate the presence of mutations in the original DNA and also confirmed disease segregation (Fig. 3e–f, Table 2, and online resource Fig. 4). Sequencing, coverage and variant statistics were similar to the previous experiment (online resource Table 4). The identified mutations in *RYR1*, *TTN* and *COL6A3* genes were in agreement with the clinical data (online resource data for patient descriptions). Importantly, while variants ranking can be made without detailed clinical and histological data to suggest probable mutated genes, such data are very valuable to validate the molecular findings. For example, we identified compound heterozygous mutations (including a nonsense mutation) in the ryanodine receptor (*RYR1)* in patient P presenting with muscular dystrophy and arthrogryposis (Fig. 3e–f). Indeed *RYR1* mutations have previously been linked to several congenital myopathies, [29] and also to severe neonatal arthrogryposis [26]. The mutations were found in his affected twin brother and each parent was found to be heterozygous for one mutation. We also identified probable mutations in the large *TTN* gene in patients J and O with myopathy with cytoplasmic aggregates and limb girdle muscular dystrophy, respectively, widening the clinical and molecular spectra for this gene that is not routinely sequenced on a diagnosis setting due to its large size.

We did not have false negative in the eight patients with known mutations as we retrieved all mutations. We checked the rate of false positive, i.e. variant not present in the starting DNA, in patients with unknown mutations where we found the probable disease-causing mutations. We found that the probability of being false positive due to sequencing or mapping errors is high when the percentage of reads showing the change is less than 25 or when the number of reads showing the change is less than 8. Even with these cut-offs, 16.5 % of false positive variants were found out of 40 variants tested, calling for validation of the mutations by Sanger sequencing.

## Discussion

In this study, we performed targeted sequencing of the coding sequences and all intron–exon boundaries including at least 50 intronic nucleotides of the known NMD genes through massively parallel sequencing in a cohort of patients with heterogeneous neuromuscular diseases. We were able to retrieve all the known mutations in previously characterized patients and we identified several novel pathogenic mutations in patients lacking molecular diagnosis.

We demonstrate that this strategy can detect several types of mutations including intronic and exonic changes as well as small indel and a large deletion. All mutations were detected from the massively parallel sequencing and analysis of a single proband, unlike previous studies where comparative sequencing of several individual exomes was used to retrieve the causative mutations in a family with HSMN [16]. A main challenge in NGS data analysis is the identification of the pathogenic change among the large list of variants. Our blind analysis based on variant ranking and disease class allowed the identification of all known mutations. However, detailed clinical, histological and molecular data were necessary for the confirmation steps, i.e., matching the genetic data with the phenotype.

Such targeted parallel sequencing of all candidate genes is especially suitable for diseases with high genetic heterogeneity, as it is the case for NMD, and should ease the identification of allelic diseases, i.e., different diseases caused by mutations of the same gene. In addition, this strategy allows the analysis of large genes, such as *TTN*, that are routinely not fully tested by conventional Sanger sequencing even if known to be implicated in diseases. For example, our identification of a probable *TTN* mutation in a patient with myopathy with cytoplasmic aggregates and respiratory insufficiency widen the clinical spectrum compared to previous studies [5].

We did not find disease-causing mutations among the coding sequences of the NMD genes in four patients with unknown genetic cause. Patient I was first clinically diagnosed with demyelinating polyneuropathy, but clinical and biochemical re-analyses in parallel to NGS suggested he had a mitochondrial disease which implicated genes are not covered by our present design. Patient N showed two missense changes in *LMNA* including the p.Arg644Cys change, previously linked to various laminopathies. Both changes are on the same allele, as they were always found in the same reads/fragments (online resource Fig. 4e), and thus cannot be the sole cause of the axonal neuropathy. We did not have access to parent DNAs to investigate this further. For the other two patients, who were previously excluded for several candidate genes by Sanger sequencing, mutations were also missed by our approach. The disease-causing mutation may be a deep intronic change, repeat expansions or translocation for which detection has not been tested in this study. Concerning specifically repeat expansions, they cannot be mapped back to a reference genome unambiguously due to their repetitive nature. Alternatively, these patients may also be mutated in a gene not linked to NMD at the time of our targeting library design.

WGS or WES would in principle allow re-analysis of variants list once newly discovered genes are identified, while these genes should be added to update the NMD capture library. However, these approaches have several disadvantages for routine molecular diagnosis compared to the *NMD*-*seq* strategy described in this study, especially concerning design, coverage, variant analysis and validation, incidental findings, and throughput and price. WES capture library should also be updated to incorporate novel gene and exon predictions, and cannot be customized to increase the enrichment of specific exons difficult to capture or incorporate known intronic mutation hotspots. *NMD*-*seq* has a higher coverage and leads to a smaller list of variants as it focuses on a subset of genes, whereas the sensitivity and heterozygosity assessment decrease following WGS or WES due to lower coverage [31]. Sequencing more genes at a lower coverage leads to an increased risk of false negative and an increased number of false positive variants that are time-consuming to validate. Indeed, WES at 50× mean coverage results in about 20 % of targeted regions covered less than 10 times, outputs not suited for routine diagnosis. WES or WGS also potentiate incidental finding, i.e. the discovery of an unrelated disease not targeted by the diagnosis measure; this could be an ethical issue. Moreover, WGS or WES have lower throughput and a higher cost, both are important issues for routine diagnosis. We validated DNA multiplexing for four DNAs in one channel to increase throughput and decrease the cost in comparison with conventional Sanger approaches which is about 500–1,000 € per sample for one gene but can increase to 5,000 € or more depending on gene size. Testing several candidates on a gene-by-gene basis may exceed 8,000 € [9]. With recent developments in sequencers and DNA barcoding, we estimate the total cost of *NMD*-*seq* from a pool of 12 barcoded DNAs to about 500 € per patient for at least 140× coverage, while WES and WGS cost about 1,500 € and 5,000 € for a 50× coverage, respectively. It takes about 2 months to perform the NMD-seq approach for 267 genes (excluding validation of the data by Sanger sequencing), a similar turnaround time to what is proposed by diagnosis laboratories to test a gene with 20 exons under current routine diagnosis.

Major conditions for further use of the *NMD*-*seq* strategy as a routine approach in genetic diagnostic labs are the reproducibility, detection sensitivity and the study of heterogeneous cohort of patients with sometimes incomplete clinical characterization as it was the case in our study. After validation of other types of mutations not tested in this study, this strategy could be implemented as a first screening approach, potentially before the need of more invasive and time-consuming investigations such as biopsies.

Our strategy does not require the knowledge of detailed clinical data for proposing candidate mutations; however, this knowledge is necessary for the final validation of the diagnosis that can only be performed matching both clinical and genetic data. NGS will not replace clinical investigations but rather direct clinicians towards the most adequate investigations, while excluding unnecessary costly and time-consuming tests. As a general rule for all NGS approaches, the targeted regions are not homogeneously covered. This point could most probably be enhanced by increasing the amount of oligonucleotides targeting regions more difficult to enrich. For an exclusion diagnosis, conventional Sanger sequencing of specific exons might be necessary if the best candidate genes for a specific disorder are not well covered by NGS. In any case, mutations found by NGS must be validated by Sanger sequencing to rule out sequence errors introduced during the NGS protocol, to provide a second independent confirmation and a basis for a simplified test for further counseling within the family. Importantly, variant ranking and confirmation depend on clinical data and knowledge on NMD, and are thus probably not directly applicable for direct-to-consumer testing.

A faster molecular diagnosis of NMD will have major impacts on patients as it will improve disease management and genetic counseling, and will allow access to therapy or inclusion into therapeutic trials. As an example, the identification of *RYR1* mutations in patient P is of major medical importance as the treatment of *RYR1* patients with salbutamol has shown significant amelioration of muscle weakness [15]. In conclusion, we provided the first proof-of-principle that next generation sequencing could be apply for molecular diagnosis of neuromuscular disorders.

## Electronic supplementary material

Below is the link to the electronic supplementary material.

Supplementary material 1 (pdf 922 kb)

Supplementary material 2 (XLS 118 kb)

Supplementary material 3 (XLS 949 kb)

Supplementary material 4 (pdf 20 kb)

Supplementary material 5 (XLS 34 kb)
