# Supplementary material for: Next generation sequencing for molecular diagnosis of neuromuscular diseases
Source: Acta Neuropathol. 2012 Apr 18;124(2):273–83. doi: 10.1007/s00401-012-0982-8 (PMC3400754; doi:10.1007/s00401-012-0982-8)
Supplement: Supplementary file 1 — Supplementary material 1 (pdf 922 kb) [file 401_2012_982_MOESM1_ESM.pdf]

ActaNeuropathologica

Next generation sequencing for molecular diagnosis of neuromuscular diseases

Nasim Vasli[1,2,3,4,5], Johann Böhm[1,2,3,4,5,$](PhD), Stéphanie Le Gras[1,2,3,4,$], Jean Muller[1,2,3,4,5,6](PhD), Cécile Pizot[1,2,3,4,5], Bernard Jost[1,2,3,4](PhD), Andoni Echaniz-Laguna[7](MD), Vincent Laugel[8](MD), Christine Tranchant[7](MD), Rafaelle Bernard[9](MD), Frédéric Plewniak[1,2,3,4](PhD), Serge Vicaire[1,2,3,4], Nicolas Levy[9](MD, PhD), Jamel Chelly[10](MD, PhD), Jean-Louis Mandel[1,2,3,4,5,6](MD, PhD), Valérie Biancalana[1,2,3,4,5,6](PhD), Jocelyn Laporte[1,2,3,4,5*](PhD).

[1]IGBMC (Institut de Génétique et de Biologie Moléculaire et Cellulaire), Illkirch, France
[2]Inserm, U964, Illkirch, France
[3]CNRS, UMR7104, Illkirch, France
[4]Université de Strasbourg, Illkirch, France
[5]Collège de France, chaire de génétique humaine, Illkirch, France
[6]Laboratoire Diagnostic Génétique, Faculté de Médecine, CHRU, Strasbourg, France
[7]Département de Neurologie, Hôpital Civil de Strasbourg, Strasbourg, France
[8]Service de Pédiatrie, Centre Hospitalier Universitaire (CHU), Strasbourg, France
[9]Faculté de Médecine de Marseille, Université de la Méditerranée, Inserm UMRS 910 Génétique Médicale et Génomique Fonctionnelle, Marseille, France
[10]Institut Cochin, INSERM Unité 1016, CNR UMR 1408, Université Paris Descartes, Sorbonne Paris Cité, Paris, France

[$]Equal contributors
Corresponding author: Dr Jocelyn Laporte

Corresponding author's address: 1, rue Laurent Fries, BP10142, 67404 Illkirch, France.
Corresponding author's phone and fax: Phone: +33 3 88653412- fax: +33 3 88653246

Corresponding author's e-mail address: jocelyn@igbmc.fr

Online Resource data

Enrichment factor

Enrichment factor for the targeted NMD genes, following targeted capture and sequencing, was calculated as the ratio between the total number of sequenced nucleotides related to the total size of the human genome versus the total number of nucleotides on target regions related to the total size of targeted regions.

Clinical and segregation data for patients I-P without previous molecular characterization

Online resource table 3 lists the sequencing data for all patients, the found mutations, the predicted amino acid change effect and the rank of the mutation among all found variants.

Patient I is a 43 year old Algerian man, born from a consanguineous marriage, with an affected sister. He first presented with polyneuropathy since he was 8 years old and he was diagnosed

with recessive demyelinating Charcot-Marie-Tooth disease (CMT4). However, additional clinical investigations showed he was affected by a mitochondrial disease. We did not find any probable mutations in the NMD genes targeted by next generation sequencing, and genes implicated in such diseases were not targeted by the *NMD-seq* capture library.

Patient J is a 56 years-old Portuguese man with isolated respiratory insufficiency since the age of 46. He had no muscle weakness, no muscle wasting, and no dysphagia. CK levels were normal. He had a sister who also presented with isolated respiratory insufficiency. Muscle biopsy demonstrated cytoplasmic body myopathy in both patients. Edstrom et al described patients with similar clinical signs and an autosomal dominant mutation in the kinase domain of TTN [1, 2]. This myopathy was called hereditary myopathy with early respiratory failure (HMERF). In our patient the probable mutation was in the *TTN* gene, thus in accordance with previous findings in these other patients. However, in our patient the mutation is outside of the kinase domain and this may explain the milder presentation of disease in this patient. In our patient, the respiratory insufficiency appeared later in life, widening the clinical severity that can be associated to dominant *TTN* mutation.

Patient K is a 35-year old French woman with a muscular dystrophy resembling either a limb girdle muscular dystrophy or Bethlem/Ullrich syndrome. She developed progressive moderate proximal and also distal muscle weakness since age 20. CK level is normal. Histological data suggested myofibrillar myopathy. Her father is affected with a milder myopathy and her brother also has difficulties to rise from a chair, that may be due to either a congenital hip dislocation or to muscle weakness. This brother has two daughters showing congenital hip dislocation and a very severe neonatal muscle weakness. Assuming they all share the same disease with different clinical expression, none of the candidate genes in patient K were found similarly mutated in all four other affected relatives. However, a probable heterozygous mutation in *COL6A3* is common to patient K, his father and his brother, all sharing similar age of onset and ambulation difficulties. This variant is absent in the unaffected mother. Mutations in the *COL6A3* gene have been previously implicated in other patients with Bethlem/Ullrich syndrome. Based on our sequencing findings, complementary clinical analyses are ongoing to determine whether the different family members suffer from the same disease with strong clinical variability, or whether two diseases segregate in the family.

Patient L is a 5-year old boy affected with familial spastic paraplegia. An affected cousin lives in Turkey and could not be examined. He has walking problems, increased deep tendon reflexes and bilateral Babinski's sign. He also shows some signs of progressive muscle weakness and amyotrophy. Cerebrospinal MRI and CK levels were normal. Muscle biopsy was refused by parents. We couldn't find any variations in genes known to be implicated in spastic paraplegias.

Patient M is a 47-year old French man with vacuolar myopathy. From 30 years old he showed muscle weakness in his legs. He is a sporadic case. We excluded all candidate variants by checking non-affected parents and sister.

Patient N is a 38-year old Algerian man from a consanguineous family. He has axonal hereditary sensorimotor neuropathies (HSMN). One brother and two sisters are affected by the same disease. Following our next generation sequencing and data analysis protocol, two probable heterozygous variations were found in the Lamin A/C gene, including the p.Arg644Cys missense

previously reported as pathogenic in different laminopathies and never found in control populations. However, both variants are on the same allele based on NGS data, as the two changes were always found in the same reads/fragments (Online resource Fig 4e), we favor the implication of a novel gene not targeted by the *NMD-seq* library. Parents DNAs were not available to investigate this further.

Patient O is a 50-year old French woman with limb girdle muscular dystrophy. She has one affected brother. She developed proximal upper and lower limbs weakness since age 35. Symptoms are stable. We found two probable heterozygous mutations in the *TTN* gene by next generation sequencing that were confirmed to be both present in her affected brother by Sanger sequencing. Her mother was carrier of one of the changes in *TTN*, while DNA from the father was not available.

Patient P is a French 13-year old boy with congenital muscular dystrophy and arthrogryposis. He has an affected twin brother. He showed congenital muscle weakness, stiff ankles and knees, feeding problems and respiratory distress at birth. He was never able to walk and later developed severe scoliosis and chronic restrictive respiratory failure. Muscle biopsy showed extensive muscle fiber degeneration and fibrosis, as well as numerous rod inclusions. We found two probable heterozygous mutations in *RYR1* in both twins, including a stop codon, and each parent was heterozygous carrier of one of the variants.

Online resource figures:

Online resource Fig 1: Sequence coverage. a) Number of non-covered exons is depicted for 1 to all 16 patients tested. Non covered exons are exons with coverage less or equal to 2x. Most non-covered exons are similar between samples and GC rich. A full list is found in online resource table 2. b) Coverage depends on GC content as increasing percentage of GC parallels with decreasing nucleotide coverage. Displays patient A data.

a

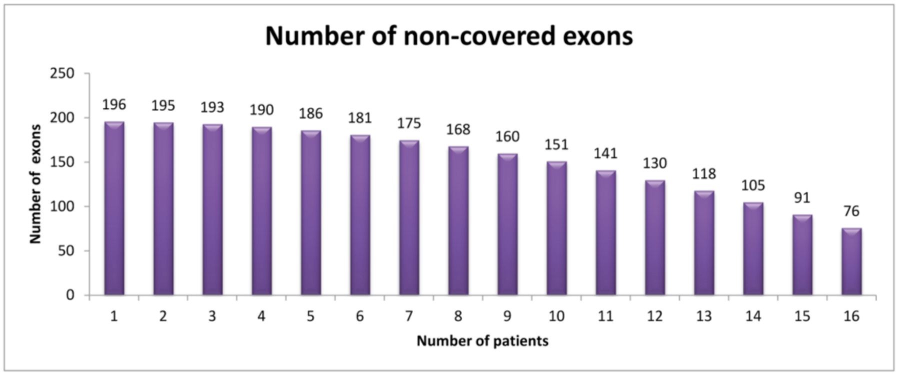

b

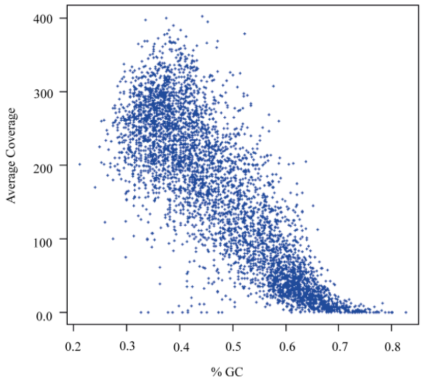

Online resource Fig 2: Coverage in targeted exons

Distribution of average coverage within exons in autosomal chromosomes across all samples tested. Coverage is not homogeneous across all regions; however, coverage is similar across samples at a given position as shown by the narrow distribution. The median has been computed based on the average coverage of all samples.

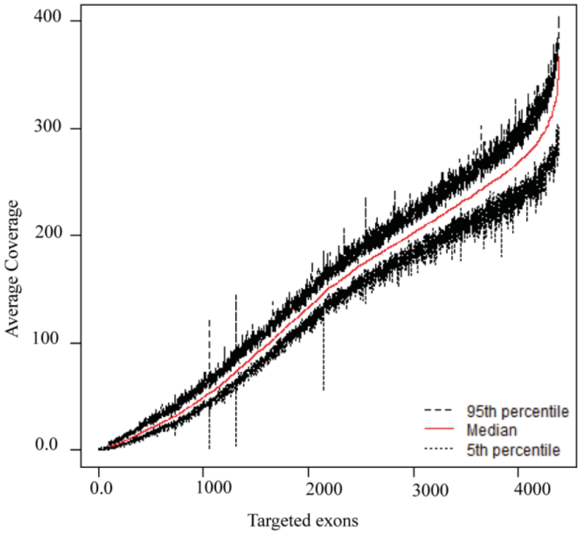

Online resource Fig 3: Detection of different types of mutations. a) Detection of an exonic heterozygous deletion in the *MTM1* gene in a female carrier for X-linked myotubular myopathy (patient A). The mutation in exon 4 is c.141-144delAAAG; p.Glu48fsX24. b) Detection of a hemizygous small insertion in the *MTM1* gene in a patient with X-linked myotubular myopathy (patient E). The mutation in exon 4 is c.156insA; p.Cys53fsX8. c and d) Detection of compound heterozygous mutations in the *SETX* gene in a patient with autosomal recessive ataxia (patient D). The mutations in exons 10 and 11 are c.3213_3214insT; p.Q1072fsX3 and c.5275-1G>A, respectively. e) Detection of a heterozygous exonic point mutation in the *DNM2* gene in a patient with autosomal dominant centronuclear myopathy (patient F). The mutation in exon 14 is c. 1565G>A, p.Arg522His. Figures displayed with the integrative genomics viewer IGV. The normal nucleotide and protein sequences are depicted at the bottom.

a *MTM1*: c.141-144delAAAG, p.Glu48LeufsX24
heterozygous deletion
patient A

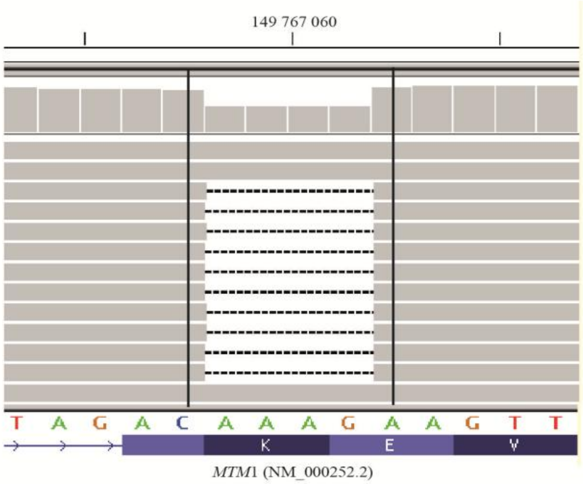

b *MTM1*: c.156-157insA, p.Cys53MetfsX8
hemizygous insertion
patient E

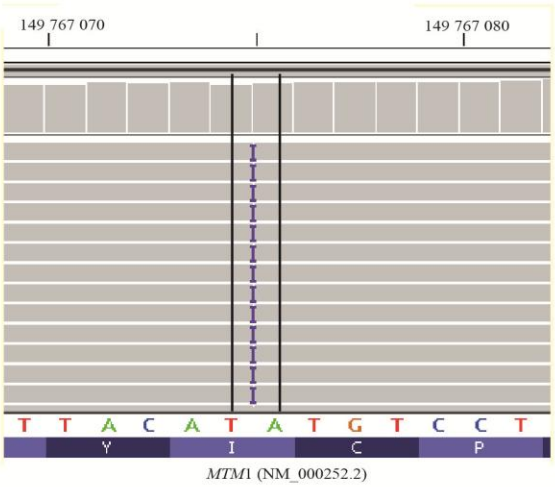

c *SETX*: c.3213-3214insT, p.Gln1072SerfsX3
heterozygous insertion
patient D

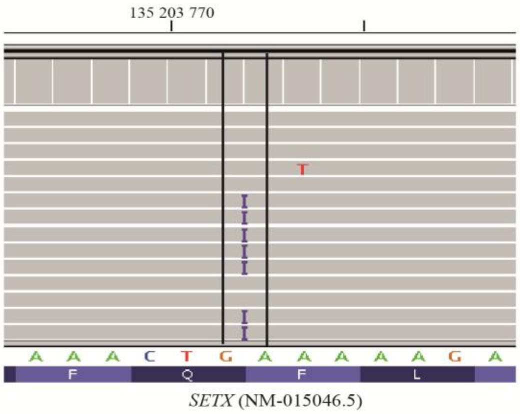

d *SETX*: c.5275-1G>A
heterozygous intronic mutation
patient D

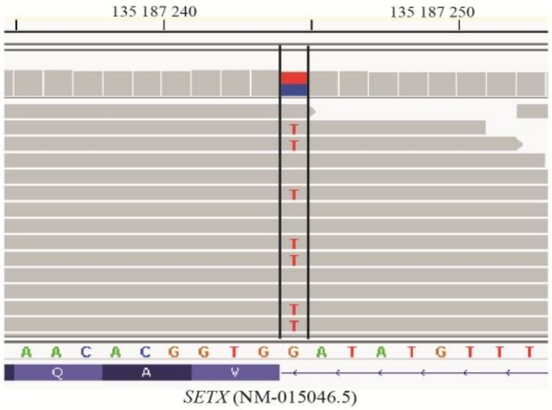

e *DNM2*: c.1565G>A, p.Arg522His
heterozygous exonic point mutation
patient F

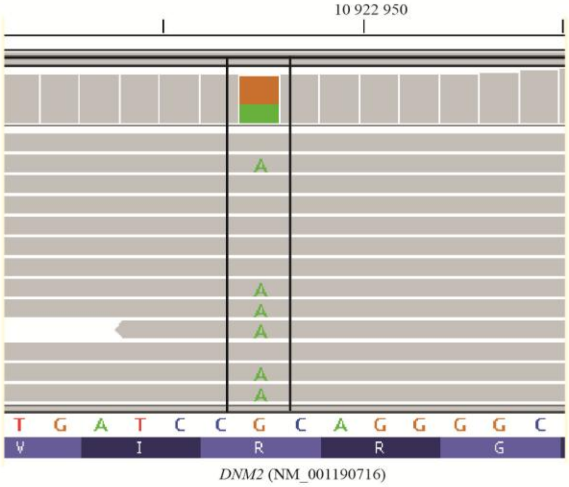

Online resource Fig 4: Detection of novel mutations. a) and b) Detection of compound heterozygous mutations in the *TTN* gene in a patient with limb girdle muscular dystrophy (patient O). The mutations in exons 18 and 240 are c.3100G>A, p.Val1034Met and c.49243G>A, p.Ala16415Thr. c) Detection of a heterozygous mutation in the *TTN* gene in a patient with myopathy with cytoplasmic aggregates (patient J). The mutations in exon 292 is c.68576C>T, p.Pro22859Leu. d) Detection of an exonic heterozygous point mutation in the *COL6A3* gene in a patient with autosomal dominant Bethlem/Ullrich syndrome (patient K). The mutation in exon 27 is c.6812G>A, p.Arg2271Lys. e) Detection of monoallelic heterozygous variations in the *LMNA* gene in a patient with axonal neuropathy (patient N). The variations in exon 11 are c.1928C>A, p.Thr643Asn and c.1930C>T, p.Arg644Cys. The p.Arg644Cys change was previously reported as pathogenic in laminopathies. Figures displayed with the integrative genomics viewer IGV. The normal nucleotide and protein sequences are depicted at the bottom.

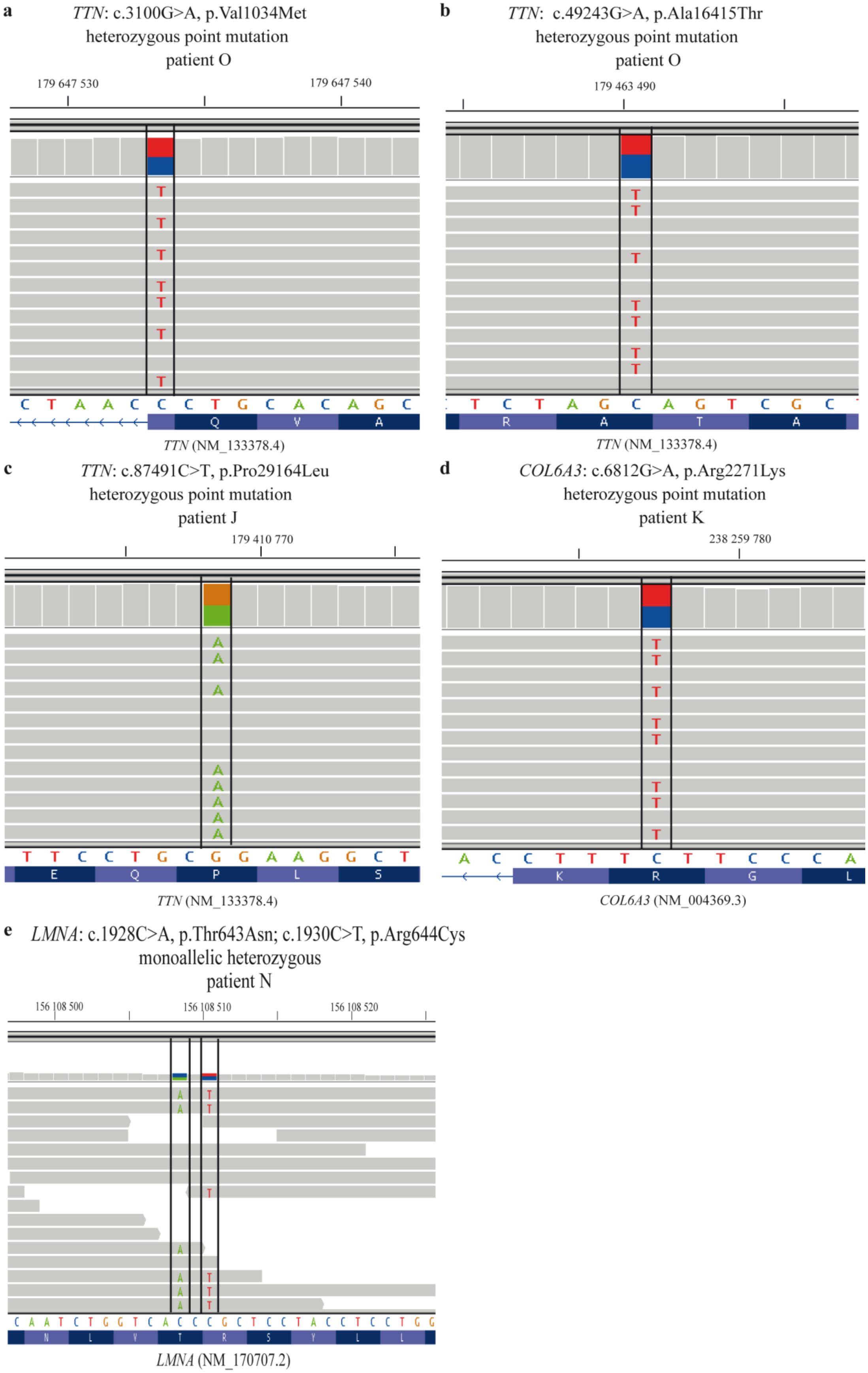
a
TTN: c.3100G>A, p.Val1034Met
heterozygous point mutation
patient O
179 647 530
179 647 540
TTN (NM_133378.4)
b
TTN: c.49243G>A, p.Ala16415Thr
heterozygous point mutation
patient O
179 463 490
TTN (NM_133378.4)
c
TTN: c.87491C>T, p.Pro29164Leu
heterozygous point mutation
patient J
179 410 770
TTN (NM_133378.4)
d
COL6A3: c.6812G>A, p.Arg2271Lys
heterozygous point mutation
patient K
238 259 780
COL6A3 (NM_004369.3)
e
LMNA: c.1928C>A, p.Thr643Asn; c.1930C>T, p.Arg644Cys
monoallelic heterozygous
patient N
156 108 500
156 108 510
156 108 520
LMNA (NM_170707.2)

Online resource Table 1: List of the 267 targeted neuromuscular disease genes and associated diseases.

Online resource Table 2: Coordinates, GC content and mean coverage of targeted exons in each patient.

Online resource Table 3: Variants ranking, conservation scores and amino acid change scores.

Online resource Table 4: Sequencing, coverage and variant statistics for patients with previously unknown mutations.

References

1. Edstrom, L., Thornell, L.E., Albo, J., Landin, S., and Samuelsson, M. (1990) Myopathy with respiratory failure and typical myofibrillar lesions. *J Neurol Sci* 96, 211-228
2. Lange, S., Xiang, F., Yakovenko, A., *et al.* (2005) The kinase domain of titin controls muscle gene expression and protein turnover. *Science* 308, 1599-1603
