# Supplementary material for: Next generation sequencing for molecular diagnosis of neuromuscular diseases
Source: Acta Neuropathol. 2012 Apr 18;124(2):273–83. doi: 10.1007/s00401-012-0982-8 (PMC3400754; doi:10.1007/s00401-012-0982-8)
Supplement: Supplementary file 4 — Supplementary material 4 (pdf 20 kb) [file 401_2012_982_MOESM4_ESM.pdf]

Supplementary Table 3: Variants ranking, conservation scores and amino acid change scores

Patient	Type of mutation	Gene	Mutation nucleotide (protein)	Sequence reads	Prediction with SIFT or polyphen	VaRank ranking for all 267 NMD genes	VaRank ranking for disease class [4]
A	Indel	*MTM1*	c.141-144delAAAG (p.Glu48LeufsX24)	125 / 87	n.a.	2	1
B	Exonic point mutation	*BIN1*	c.1717C>T (p.Gln573X)	0 / 11	n.a.	3	1
C	Large deletion	*DMD*	Deletion ex18-44	n.a.	n.a.	large deletion found	large deletion found
D	Indel + intronic splice site mutation	*SETX*	c.3213-3214insT (p.Gln1072SerfsX3); c.5275-1 G>A	129 / 102; 66 / 67	n.a. - n.a.	2 [2]	1 [2]
E	Indel	*MTM1*	c.156-157ins A (p.Cys53MetfsX8)	6 / 96 [3]	n.a.	2	1
F	Exonic point mutation	*DNM2*	c.1565 G>A (p.Arg522His)	25 / 17	Probably damaging	7	1
G	Intronic, effect on splice	*MTM1*	c.1261-10A>G	0 / 80	n.a.	28	1
H	Indel + exonic point mutation	*SETX*	c.2967-2971delGAAAG (p.Arg989SerfsX5); c.994 C>T (p.Arg332Trp)	57 / 116; 74 / 161	n.a. - Deleterious	2	1 [2]
I	not found	none			n.a.		
J	Exonic point mutation	*TTN*	c.68576C>T (p.Pro22859Leu)	137/131	Deleterious	3	1
K	Exonic point mutation	*COL6A3*	c.6812G>A (p.Arg2271Lys)	37 / 36	Possibly damaging	28	3 [5]
L	not found	none			n.a.		
M	not found	none			n.a.		
N	Exonic point mutation	none (LMNA)	c.1928C>A (p.Thr643Asn); c.1930C>T (p.Arg644Cys 2)	13 / 11; 13 / 11	Deleterious - Deleterious	13	1 [2]
O	Exonic point mutation	*TTN*	c.3100G>A (p.Val1034Met); c.49243G>A (p.Ala16415Thr)	162 / 131; 72 / 75	Deleterious - Deleterious	7	1 [2]
P	Exonic point mutation	*RYR1*	c.8554C>T (p.Arg2852X); c.11557G>A (p.Glu3853Lys)	69 / 45; 23 / 34	n.a. - Deleterious	2 [2]	1 [2]

[1] reads for WT/mutant allele

[2] in a recessive scenario

[3] 94% reads show the variant and the heterozygous limit was set to 80%
